# Significance of I313V mutation of NLPR3 gene in two pediatric patients

**DOI:** 10.1186/1546-0096-9-S1-P305

**Published:** 2011-09-14

**Authors:** A Omenetti, R Antona, R Gallizzi, M Alessio, M Finetti, S Carta, A Rubartelli, A Martini, M Gattorno

**Affiliations:** 1 Department of Pediatrics, G Gaslini Institute, Genoa; 2 Department of Pediatrics, University of Palermo; 3 Department of Pediatrics, University of Messina; 4 Department of Pediatrics, University of Naples; 5 Cell Biology Unit, National Cancer Research Institute, Genoa; 6 Department of Pediatrics, University of Genoa

## Background

The I313V mutation of the NLRP3 gene has been only anecdotally reported and described in association with the so-called Magic Syndrome (Infevers database). However, nor the clinical or pathophysiological significance of such mutation has been so far reported.

## Aim

To describe the clinical picture of patients carrying the I313V mutation and its consequences in IL-1β secretion.

## Methods

Two families carrying NLRP3 I313V mutation were evaluated. Monocytes were obtained from patients and their parents. Cells isolated from healthy donors (HD) (N=14) were used as negative control group. Pattern of secretion of IL-1β, IL-1Ra, IL-6 and IL-8 were then assessed by ELlSA in the presence or absence exogenous LPS.

## Results

Both case #1 (M.T) and #2 (V.C) displayed a mild clinical phenotype (episodes of urticarial rash and arthralgia associated with elevation of acute phase reactants), compatible with FCAS and Muckle-Wells syndrome, respectively. Both patients displayed good response to NSAID and/or steroid on demand. Compared to HD controls, patients displayed enhanced and delayed IL1β secretion. This was accompanied by higher levels of lL1Ra and IL-6 without any significant differences in IL-8. Interestingly, parents carrying the mutation also displayed higher levels of secreted IL-1β compared to HD control group.

## Conclusion

The I313V mutation is associated with a mild CAPS phenotype and with an increased IL-1β secretion.

